# Proof-of-Concept Support for the Development and Implementation of a Digital Assessment for Perinatal Mental Health: Mixed Methods Study

**DOI:** 10.2196/27132

**Published:** 2021-06-04

**Authors:** Nayra Anna Martin-Key, Benedetta Spadaro, Thea Sofie Schei, Sabine Bahn

**Affiliations:** 1 Cambridge Centre for Neuropsychiatric Research Department of Chemical Engineering and Biotechnology University of Cambridge Cambridge United Kingdom; 2 Psyomics Ltd Cambridge United Kingdom

**Keywords:** COM-B, COVID-19, digital mental health, maternal mental health, paternal mental health, perinatal mental health, mental health, support, development, implementation, assessment, mother, women

## Abstract

**Background:**

Perinatal mental health symptoms commonly remain underdiagnosed and undertreated in maternity care settings in the United Kingdom, with outbreaks of disease, like the COVID-19 pandemic, further disrupting access to adequate mental health support. Digital technologies may offer an innovative way to support the mental health needs of women and their families throughout the perinatal period, as well as assist midwives in the recognition of perinatal mental health concerns. However, little is known about the acceptability and perceived benefits and barriers to using such technologies.

**Objective:**

The aim of this study was to conduct a mixed methods evaluation of the current state of perinatal mental health care provision in the United Kingdom, as well as users’ (women and partners) and midwives’ interest in using a digital mental health assessment throughout the perinatal period.

**Methods:**

Women, partners, and midwives were recruited to participate in the study, which entailed completing an online survey. Quantitative data were explored using descriptive statistics. Open-ended response data were first investigated using thematic analysis. Resultant themes were then mapped onto the components of the Capability, Opportunity, and Motivation Behavior model and summarized using descriptive statistics.

**Results:**

A total of 829 women, 103 partners, and 90 midwives participated in the study. The provision of adequate perinatal mental health care support was limited, with experiences varying significantly across respondents. There was a strong interest in using a digital mental health assessment to screen, diagnose, and triage perinatal mental health concerns, particularly among women and midwives. The majority of respondents (n=781, 76.42%) expressed that they would feel comfortable or very comfortable using or recommending a digital mental health assessment. The majority of women and partners showed a preference for in-person consultations (n=417, 44.74%), followed by a blended care approach (ie, both in-person and online consultations) (n=362, 38.84%), with fewer participants preferring online-only consultations (n=120, 12.88%). Identified benefits and barriers mainly related to physical opportunity (eg, accessibility), psychological capability (eg, cognitive skills), and automatic motivation (eg, emotions).

**Conclusions:**

This study provides proof-of-concept support for the development and implementation of a digital mental health assessment to inform clinical decision making in the assessment of perinatal mental health concerns in the United Kingdom.

## Introduction

The perinatal period, comprising pregnancy along with one year after giving birth, represents one of the most complex and challenging times in a woman’s life, with perinatal mental health disorders affecting around 15%-20% of women in the general population [1] and up to 40% of women in intensive perinatal care units [2]. Common psychiatric complications during the perinatal period include depression and anxiety [3]. Other less common conditions include new onset or recurring bipolar disorder, schizophrenia, and other psychotic illnesses, with most psychotic episodes occurring within the first 2 postnatal weeks [4]. Mental health concerns during the perinatal period do not only affect women but can also affect their partners, with approximately 5%-10% of fathers experiencing depression [5] and 5%-15% experiencing anxiety [6].

Despite these figures, perinatal mental health concerns commonly remain underdiagnosed and undertreated in maternity care settings in the United Kingdom [7]. This is likely due to a variety of reasons, including stigma and discomfort about discussing one’s own mental health, as well as a fear of consequences of disclosure [8]. Other challenges relate to a lack of mental health training and formal education, particularly among midwives, time constraints, a pressure to prioritize physical over mental health [9], and a lack of knowledge regarding available referral pathways [10].

The current long-term costs of unidentified perinatal mental health concerns to society are estimated at around £8.1 billion (US $10.1 billion) for each 1-year cohort of births in the United Kingdom, of which 72% relate to adverse impacts on the child [7]. Critically, it is estimated that this figure will be astronomically higher due to the ongoing COVID-19 pandemic. Indeed, preliminary research on COVID-19 has indicated a significant increase in psychological distress for expectant mothers [11], with strict lockdown measures disrupting routine clinical appointments and access to mental health services, leaving many at an increased risk of poor mental health [12]. These measures have also led to increased financial difficulties, higher rates of domestic violence, as well as impaired support from family and friends, all of which have been identified as risk factors for perinatal mental health difficulties [13].

In light of the recently published recommendations by the Royal College of Obstetricians and Gynaecologists [13] on the use of remote means to provide support to women throughout the perinatal period, digital technologies may offer an innovative way to support the mental health needs of women and their families throughout and beyond these trying times. Notably, digital technologies have the potential to support midwives in the recognition of perinatal mental health concerns and patients’ treatment needs. Further, research has demonstrated that individuals are more likely to report severe symptoms on technology platforms than to a health care professional (HCP) [14], and patients value the independence and empowerment that can be obtained via the use of a digital platform [15]. However, little is known about the acceptability and perceived benefits and barriers to using digital technologies in perinatal mental health care, particularly among women and partners at different stages of parenthood, as well as midwives.

The present mixed methods study aimed to examine the current state of perinatal mental health care provision in the United Kingdom, as well as in the context of the evolving pandemic, relative to the recommendations put forward by the National Institute for Health and Care Excellence (NICE) [1]. Further, we explored users’ (women and partners) and midwives’ attitudes toward using a digital mental health assessment to screen, diagnose, and triage perinatal mental health concerns. To this end, we analyzed participants’ attitudes in light of the Capability, Opportunity, and Motivation Behavior (COM-B) model, a theory of behavior that poses behavior as a result of the interaction between distinct behavioral components, namely capability, opportunity, and motivation [16]. The COM-B model has been commonly employed to design health care interventions and understand complex behaviors that result from the interplay between different stakeholders (eg, patients, carers, and health care providers) [17-19]. In our study, the model acts as a theoretical framework, informing future strategies that can address the barriers and facilitators to the uptake of a digital mental health assessment.

The ultimate goal of this research was to provide proof-of-concept support for the development and implementation of a digital mental health assessment tool to inform clinical decision making regarding the diagnosis and treatment of perinatal mental health concerns in the United Kingdom.

## Methods

### Participants

Participants were recruited between April and August 2020 via email, posts on the Facebook and Twitter pages of the Cambridge Centre for Neuropsychiatric Research, and paid Facebook advertisements. All participants provided informed consent electronically to participate in the study, which was approved by the University of Cambridge Human Biology Research Ethics Committee (approval number PRE.2020.041). Inclusion criteria for the study were: (1) ≥18 years, (2) UK residence, and (3) fluency in English. Midwives were also required to be currently practicing in the United Kingdom, while women and partners were required to fall into one of the following subgroups: (1) currently planning or trying to conceive, (2) currently pregnant or partner of someone who is currently pregnant, or (3) given birth within the last 2 years or partner of someone who has given birth within the last 2 years. There were no other inclusion criteria.

The women and partners were invited to enter their email for the chance to win a £50 (US $67) Highstreet voucher, while midwives were provided with a £15 (US $20) Highstreet voucher for their time. Of the partners who participated in the study, 65.05% (n=67) were recruited via participating women, while midwives were recruited separately, meaning that they were not necessarily involved in the care of the participating women.

### Materials and Procedure

Three anonymous online surveys were created using Qualtrics in order to explore the current state of perinatal mental health provision in the United Kingdom, as well as attitudes toward using a digital mental health assessment to screen, diagnose, and triage perinatal mental health concerns. All questions were developed in consultation with the senior author (SB), a practicing psychiatrist. The surveys were adaptive in nature, such that only relevant questions were asked based on previous responses.

The women and partner surveys comprised two separate surveys that could be completed in 10-15 minutes and included five sections: (1) sociodemographic information, (2) perinatal health information, (3) mental health care provision, (4) mental health symptoms, (5) interest in a digital mental health assessment, and (6) perceived benefits and barriers to using a digital mental health assessment. The women’s survey also included an additional section on COVID-19 and mental health (only data from women who reported being pregnant when completing the survey or who had given birth within the last 3 months and had been in contact with a midwife since the United Kingdom entered its first lockdown [end of March to beginning of July 2020] were collected). The questions were assessed for relevance, appropriateness, and length by 2 women and 2 partners. Table S1 in Multimedia Appendix 1 provides the questions included in the surveys.

The midwives’ survey could be completed in 20-30 minutes and comprised five sections: (1) sociodemographic information, (2) mental health provision, (3) partners’ mental health care provision, (4) COVID-19 and perinatal mental health care provision, (5) interest in a digital mental health assessment, and (6) perceived benefits and barriers to using a digital mental health assessment. The questions were assessed for relevance, appropriateness, and length by a practicing midwife and a practicing specialist perinatal psychiatrist. Table S1 in Multimedia Appendix 2 provides the questions included in the survey.

### Data Analytic Strategy

The processing and analysis of quantitative data (ie, frequencies and percentages) was conducted in R, version 4.0.2 (R Foundation for Statistical Computing) [20]. Figures were created using the R packages *ggplot2* and *likert*, versions 3.3.2 and 1.3.5, respectively, as well as Excel, version 16.43 (Microsoft Corp). These data included sociodemographic information, perinatal health characteristics, mental health provision, mental health symptoms, mental health and COVID-19, as well as attitudes toward the use of a digital mental health assessment throughout the perinatal period.

Qualitative data were analyzed following the recommended six stages of thematic analysis [21]. These data comprised open-ended responses regarding the perceived benefits and barriers to using a digital mental health assessment throughout the perinatal period. The first author (NAM-K) became familiar with the data by reading and rereading the open-ended responses and noting down ideas. Initial codes were then generated, allocated, and incorporated into a coding scheme, encompassing brief descriptions for each code. The second author (BS) performed a blinded allocation of codes to each of the open-ended responses using the coding scheme. Following this, any inconsistencies were discussed until a consensus was reached. The codes were then condensed into broad themes, with the first and second authors generating themes separately. These were then discussed until a consensus was reached.

The COM-B [16] was then used to condense themes further into the model’s framework and components, namely: Capacity (components: physical and psychological capacity), Opportunity (components: physical and social opportunity), and Motivation (components: automatic and reflective motivation). The COM-B model proposes that individuals need capability (C), opportunity (O) and motivation (M) to engage in a particular behavior (B). In the model, capability is defined as an individual’s ability to physically and psychologically perform a behavior of interest, opportunity indicates the external factors that can affect engagement in said behavior, and motivation refers to a construct comprising automatic and reflective drives. This model has been commonly used to design interventions and understand behaviors in clinical and public health contexts (eg, [22-26]). To map themes onto the COM-B model, both the first and second authors allocated a COM-B component to each of the established themes under blinded conditions. Once again, any inconsistencies were discussed until a consensus was reached. Frequencies were calculated per group status (ie, women, partners, midwives), with between-group comparisons assessed using the Fisher exact test. Post hoc pairwise comparisons (ie, women vs partners, women vs midwives, and partners vs midwives) were conducted using the Fisher exact test where appropriate.

## Results

### Sociodemographic Characteristics

A total of 829 women (planning or trying to conceive: n=76; currently pregnant: n=259; had given birth within the last 2 years: n=494), 103 partners (planning or trying to conceive: n=11; partner of someone who is currently pregnant: n=38; partner of someone who has given birth within the last 2 years: n=54), and 90 midwives participated in the study.

Women’s and partners’ sociodemographic information can be found in Table S1A (Multimedia Appendix 1). The average ages of the women and partners were 31.78 (SD 4.63) years and 34.87 (SD 6.38) years, respectively, with the majority of respondents being White. Over 80% (n=687) of women and approximately 75% (n=77) of partners had at least an undergraduate degree, and the majority of respondents were married or in a civil partnership. Across the women and partner groups, around 71% (n=659) owned their home and approximately 78% (n=724) had a household income of at least £35,001 (US $46,950) before tax.

Midwives’ sociodemographic information can be found in Table S1A (Multimedia Appendix 2). The average age of the midwives was 39.90 (SD 9.67) years, with the majority being female and having an undergraduate degree. The average number of years of practice was 10.36 (SD 7.19) years.

### Perinatal Health Characteristics

**Figure 1:**
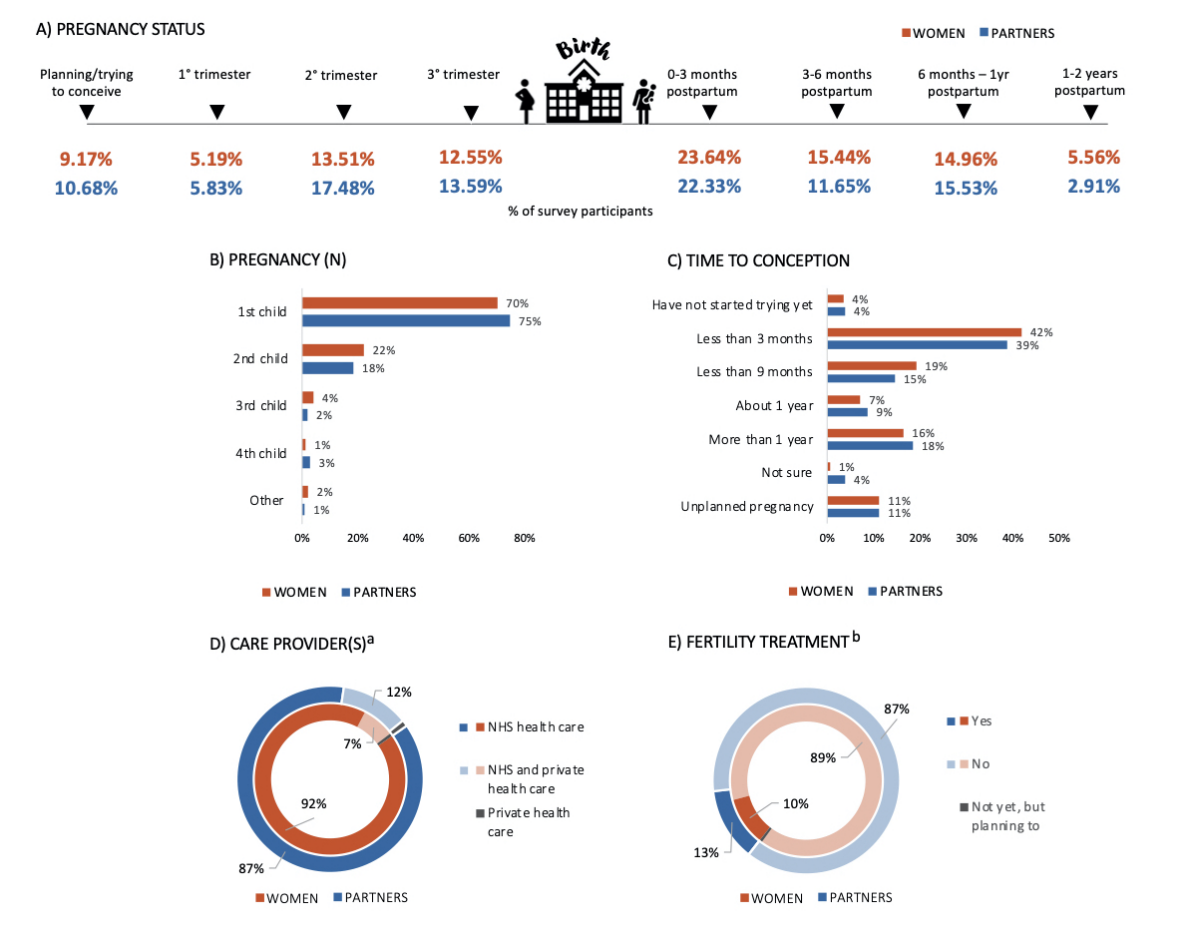
Perinatal health characteristics of women and partners, including pregnancy status (A), pregnancy number (B), time to conception (C), care provider(s) (D), and fertility treatment (E). a: Includes those in contact with the health care system (women: n=785; partners: n=93); b: Includes those who have started trying to conceive and planned pregnancies (women: n=707; partners: n=87). NHS: National Health Service.

Perinatal health characteristics for women and partners can be found in Figure 1 and Table S1B (Multimedia Appendix 1). Over half of the respondents had given birth or had a partner who had given birth within the last 2 years (Figure 1A). For the majority of respondents, this was or would be their first child (Figure 1B) and conception had occurred within 1 year (Figure 1C). The vast majority of respondents’ pregnancy or partner’s pregnancy was solely monitored by the National Health Service (NHS) (Figure 1D), and the majority of respondents had not received any fertility treatment (Figure 1E). A small percentage of women (n=49, 6.93%) and partners (n=14, 16.09%) expressed having had to terminate the pregnancy (eg, abortion, ectopic pregnancy, other medical intervention). Just under a quarter of respondents had a miscarriage (n=157, 22.21%) or had a partner who had a miscarriage (n=16, 18.39%), while around half of the respondents had experienced a difficult birth (n=262, 53.04%) or had a partner who had experienced a difficult birth (n=26, 48.15%).

### Mental Health Care Provision

**Figure 2:**
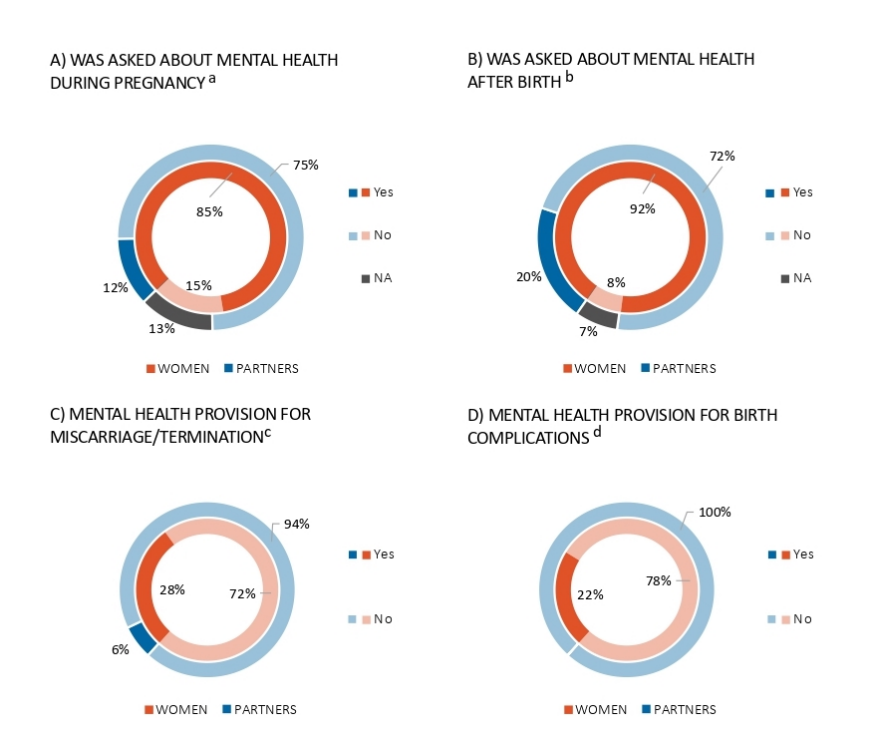
Perinatal mental health provision for women and partners, including being asked about their mental health during pregnancy (A) and after giving birth (B), as well as being offered mental health support following a miscarriage or termination (C) and after a difficult birth (D). a: Includes those who are pregnant or have given birth (women: n=753; partners: n=92); b: Includes those who have given birth (women: n=494; partners: n=54); c: Includes those who answered “yes” to the termination or miscarriage question (women: n=159; partners: n=16); d: Includes those who answered “yes” to birth complications question (women: n=262; partners: n=26). NA: not applicable.

Women’s and partners’ reported experiences with mental health care provision throughout the perinatal period can be found in Figure 2 and Table S1C (Multimedia Appendix 1). Over two-thirds of women (n=576, 73.38%) and 16.13% (n=15) of partners had received information on mental health during antenatal or postnatal appointments, with information typically provided in the form of face-to-face discussions and leaflets. The majority of women were asked about their mood or mental health at least once throughout their pregnancy or after giving birth, while the opposite was the case for partners (Figure 2A and B). Just over a quarter of women were offered mental health support or advice following a miscarriage and/or termination (Figure 2C), while 22.14% (n=58) received mental health support or advice after a difficult birth (Figure 2D). Only 1 partner (6.25%) was offered mental health support or advice following their partner’s miscarriage or termination, and none were offered any mental health support or advice after their partner’s difficult birth (Figure 2C and D).

**Figure 3:**
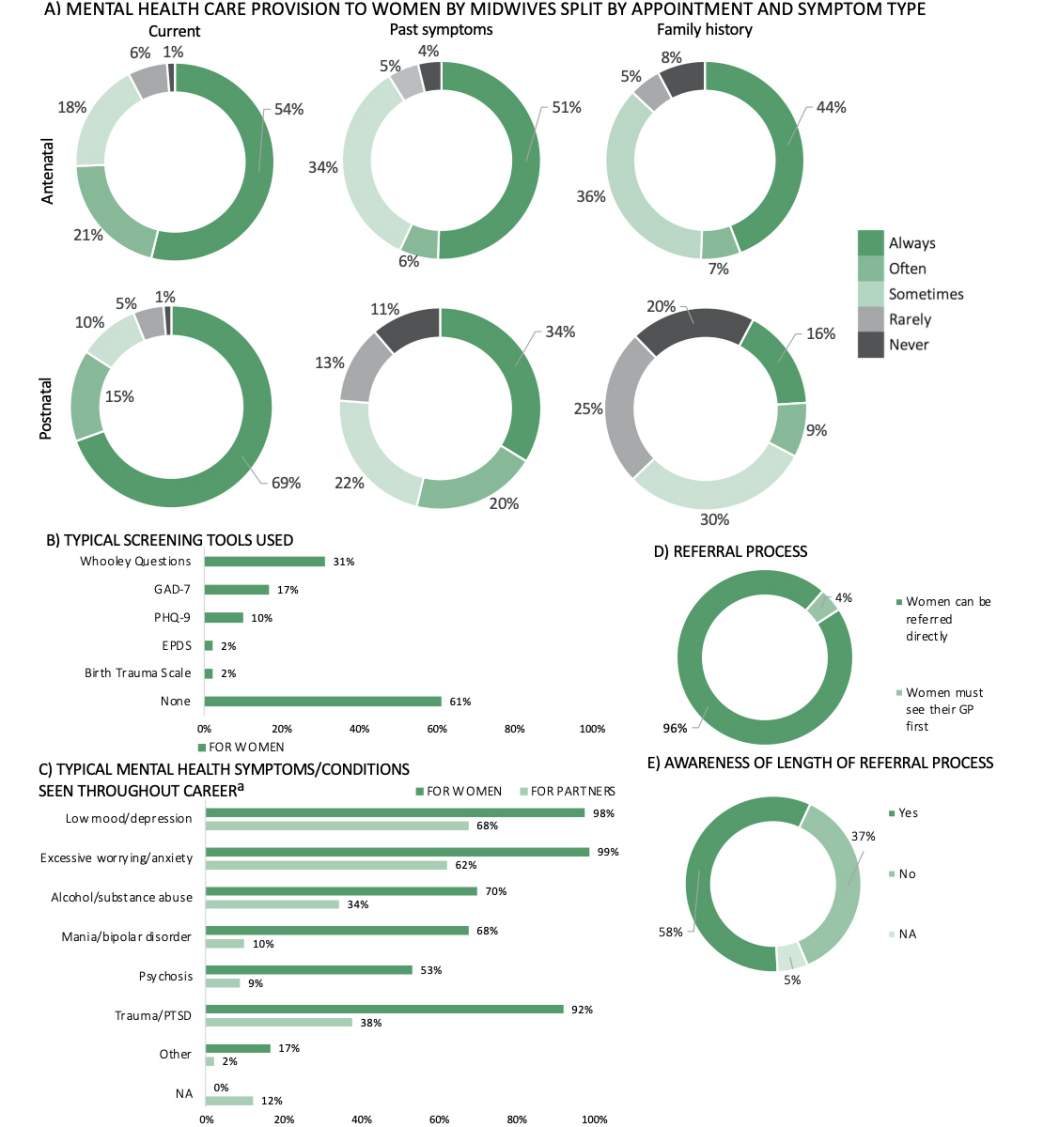
Midwives’ provision of perinatal mental health care, including mental health care provision provided to women split by appointment type (ie, antenatal and postnatal) and symptom type (ie, current, past, and family history) (A), typical mental health screening tools used (B), typical mental health symptoms or conditions encountered throughout their career (C), referral process (ie, whether patients can be directly referred to a mental health specialist or whether they have to see their general practitioner first (GP) (D), and awareness of the referral process (E). a: Percentages add to more than 100% as midwives could select multiple options. GAD-7: Generalized Anxiety Disorder-7, PHQ: Patient Health Questionnaire, EPDS: Edinburgh Postnatal Depression Scale, PTSD: posttraumatic stress disorder, NA: not applicable.

A summary of midwives’ experiences providing perinatal mental health care can be found in Figure 3 and Table S1B and S1C (Multimedia Appendix 2). One-third of midwives (n=30, 33.33%) reported always providing women with information on mental health during antenatal or postnatal appointments, with information typically provided during face-to-face appointments and via leaflets (Table S1B, Multimedia Appendix 2). Approximately 50% (n=42) of midwives reported always asking patients about their current mental health symptoms throughout the antenatal period, while almost two-thirds (n=57) expressed always asking patients about their current mental health symptoms throughout the postnatal period (Figure 3A). These figures were lower when inquiring about past symptoms and a family history of mental health, throughout both the antenatal and postnatal periods (Figure 3A). The vast majority of midwives (n=55, 61.11%) used nonstandardized questions to assess patients’ mental health symptoms, while just under one-third (n=28, 31.11%) used the Whooley Questions, and fewer used tools such as the Patient Health Questionnaire-9 (PHQ-9) [27] (n=9, 10.00%) and the 7-item Generalized Anxiety Disorder questionnaire (GAD-7) [28] (n=15, 16.67%) (Figure 3B). The most common mental health conditions encountered were depression, anxiety, and trauma or posttraumatic stress disorder (PTSD) (Figure 3C). The majority of midwives (n=86, 95.56%) reported being able to directly refer a patient to a mental health specialist, with just over a half (n=52, 57.78%) being aware of the length of the referral process (Figure 3D and E).

Approximately 7% (n=6) of midwives reported always providing partners with information on mental health (Table S1C, Multimedia Appendix 2). When provided, mental health information was typically made available during face-to-face appointments (n=56, 87.50%) (Table S1C, Multimedia Appendix 2). Similar to the women, the most common mental health conditions seen in partners were depression, anxiety, and trauma or PTSD (Figure 3C).

### Mental Health Symptoms and Diagnosis

**Figure 4:**
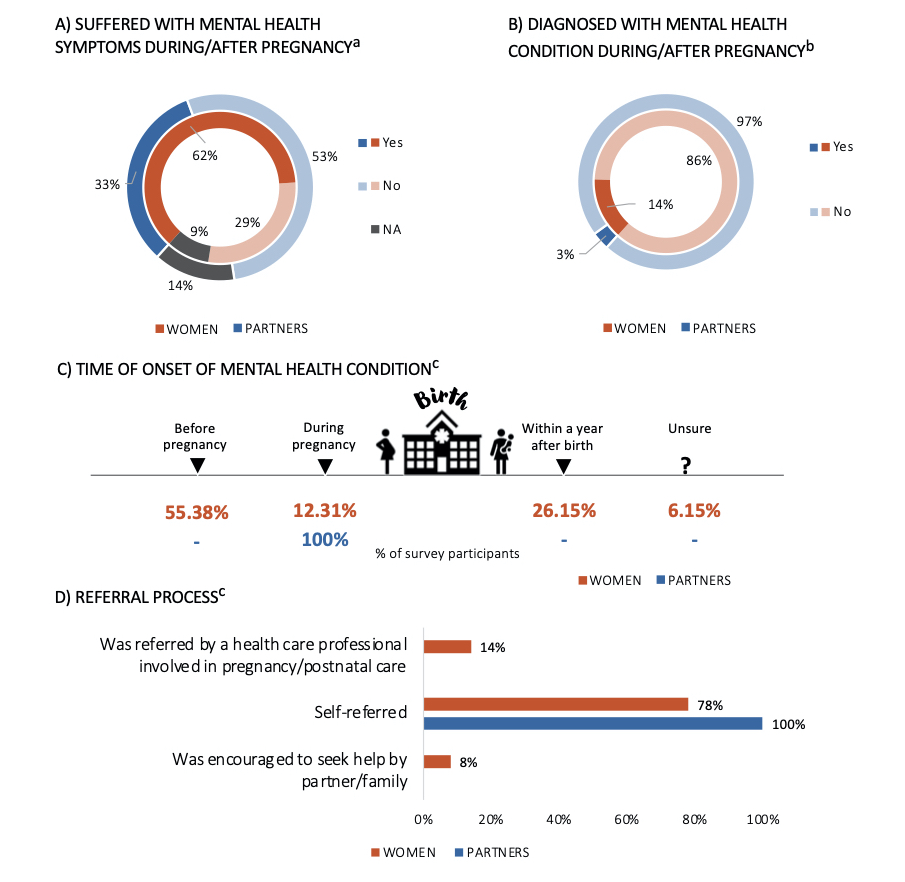
Mental health symptoms and diagnoses for women and partners, including whether they had experienced mental health symptoms during or after the pregnancy (A), whether they had been diagnosed with a mental health condition during or after the pregnancy (B), the time of onset of the mental health condition (C), and information regarding the referral process (D). a: Includes those who are pregnant or have given birth (women: n=753; partners: n=92); b: Includes those who answered “yes” to experiencing mental health symptoms during or after pregnancy (women: n=469; partners: n=30); c: Includes those who were diagnosed with a mental health condition (women: n=65; partners: n=1). NA: not applicable.

A summary of the women’s and partners’ mental health symptoms and diagnoses throughout the perinatal period can be found in Figure 4 and Table S1D (Multimedia Appendix 1). Almost two-thirds of women (n=469, 62.28%) and one-third of partners (n=30, 32.61%) reported having experienced mental health symptoms during the pregnancy and/or after delivery (Figure 4A), with approximately 13.86% (n=65) of women and around 3.33% (n=1) of partners being diagnosed with a mental health condition by a health care professional (eg, general practitioner or psychiatrist) during the perinatal period (Figure 4B). The most common diagnoses were depression and anxiety (Table S1D, Multimedia Appendix 1). Over one half of women (n=36, 55.38%) reported having had the condition before they became pregnant, while 26.15% (n=17) developed the condition within 1 year after giving birth (Figure 4C). The vast majority of respondents had referred themselves to a mental health specialist, while approximately 14% of women were referred to a mental health professional by a midwife or HCP involved in their antenatal or postnatal care (Figure 4D).

### COVID-19 and Mental Health

**Figure 5:**
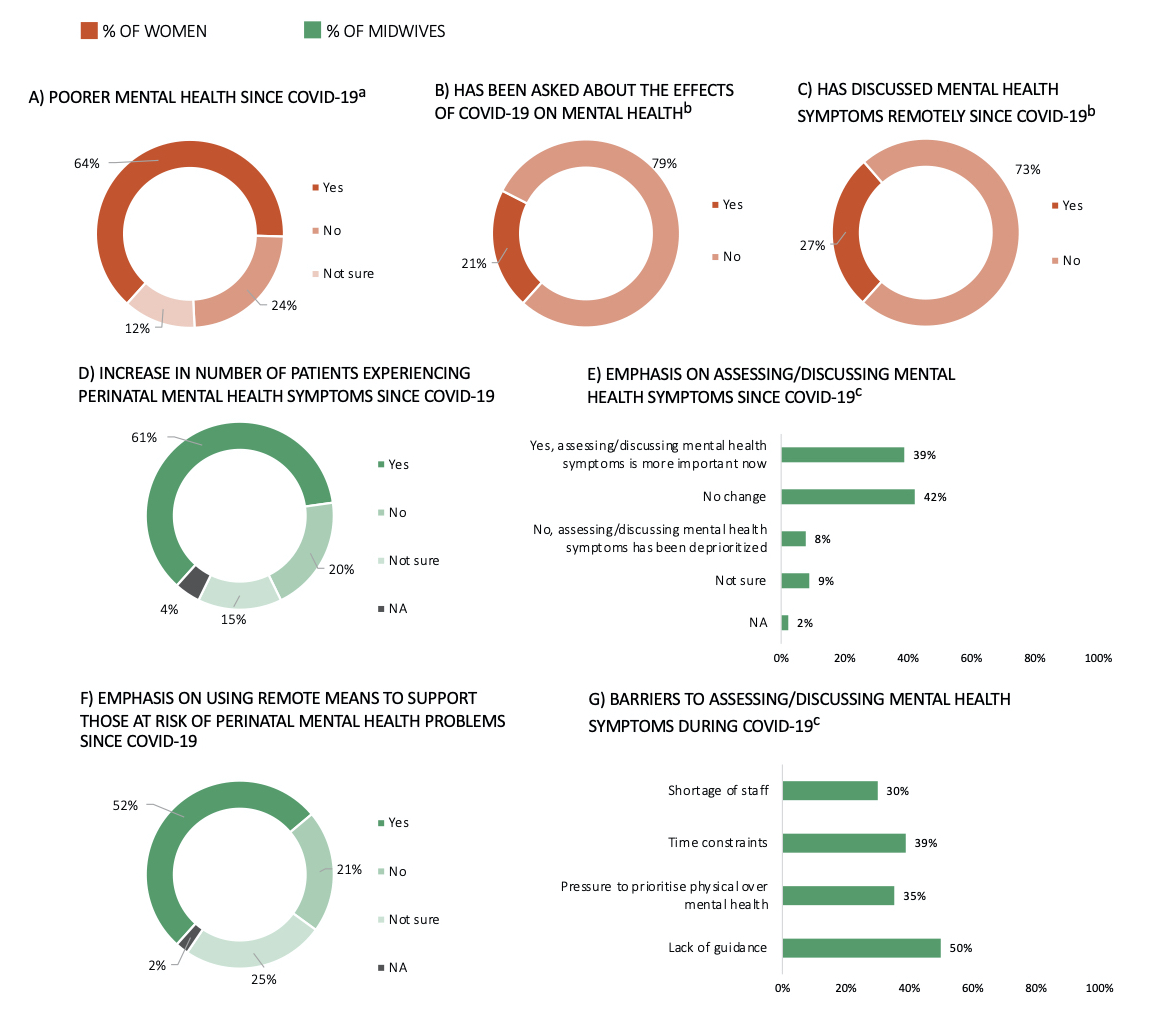
The effects of COVID-19 on women’s mental health and midwives’ mental health care provision, including the extent to which women had experienced poorer mental health since the outbreak (A), whether they had been asked about the effects of the pandemic on their mental health (B), and whether remote means (ie, telephone or video consultations) had been used to discuss their mental health throughout the pandemic (C). Regarding midwives’ mental health care provision, this included the extent to which midwives had seen an increase in the number of patients experiencing mental health difficulties since COVID-19 (D), whether there had been an emphasis on assessing or discussing mental health symptoms throughout the pandemic (E), whether there had been an emphasis on using remote means (ie, telephone or video consultations) to support those at risk of perinatal mental health difficulties since COVID-19 (F), and the barriers to assessing or discussing mental health difficulties throughout the pandemic (G). a: Includes women who are pregnant or have given birth within the last 3 months (n=455); b: Includes women who are pregnant or have given birth within the last 3 months and have been in contact with a midwife since the lockdown (n=446); c: Percentages add to more than 100% as midwives could select multiple options. NA: not applicable.

A summary of the effects of COVID-19 on women’s mental health can be found in Figure 5 and Table S1E (Multimedia Appendix 1). Almost two-thirds of women (n=293, 64.40%) reported poorer mental health symptoms following the COVID-19 outbreak (Figure 5A). Approximately 21% of women (n=93) were specifically asked about the effects of the evolving pandemic on their mental health (Figure 5B), while nearly 27% (n=120) discussed their mental health remotely (ie, via telephone or video consultation) with a midwife or HCP involved in their antenatal or postnatal care (Figure 5C).

The effects of COVID-19 on midwives’ experiences providing mental health care are summarized in Figure 5 and Table S1D in Multimedia Appendix 2. A total of 55 (61.11%) midwives reported seeing an increase in perinatal mental health symptoms since the COVID-19 outbreak (Figure 5D). The majority of midwives reported there being no change to the standard of mental health care, although this was closely followed by an emphasis on prioritizing asking about mental health (Figure 5E). Further, 52.22% (n=47) of respondents reported using remote means (ie, telephone or video consultation) to discuss patients’ mental health symptoms (Figure 5F), and 50% (n=45) of midwives saw a lack of guidance on how to evaluate mental health symptoms throughout the pandemic as an important barrier to assessing perinatal mental health. Other important barriers were time constraints and a pressure to prioritize physical over mental health (Figure 5G).

### Interest in a Digital Mental Health Assessment to Screen, Diagnose, and Triage Perinatal Mental Health Symptoms

**Figure 6:**
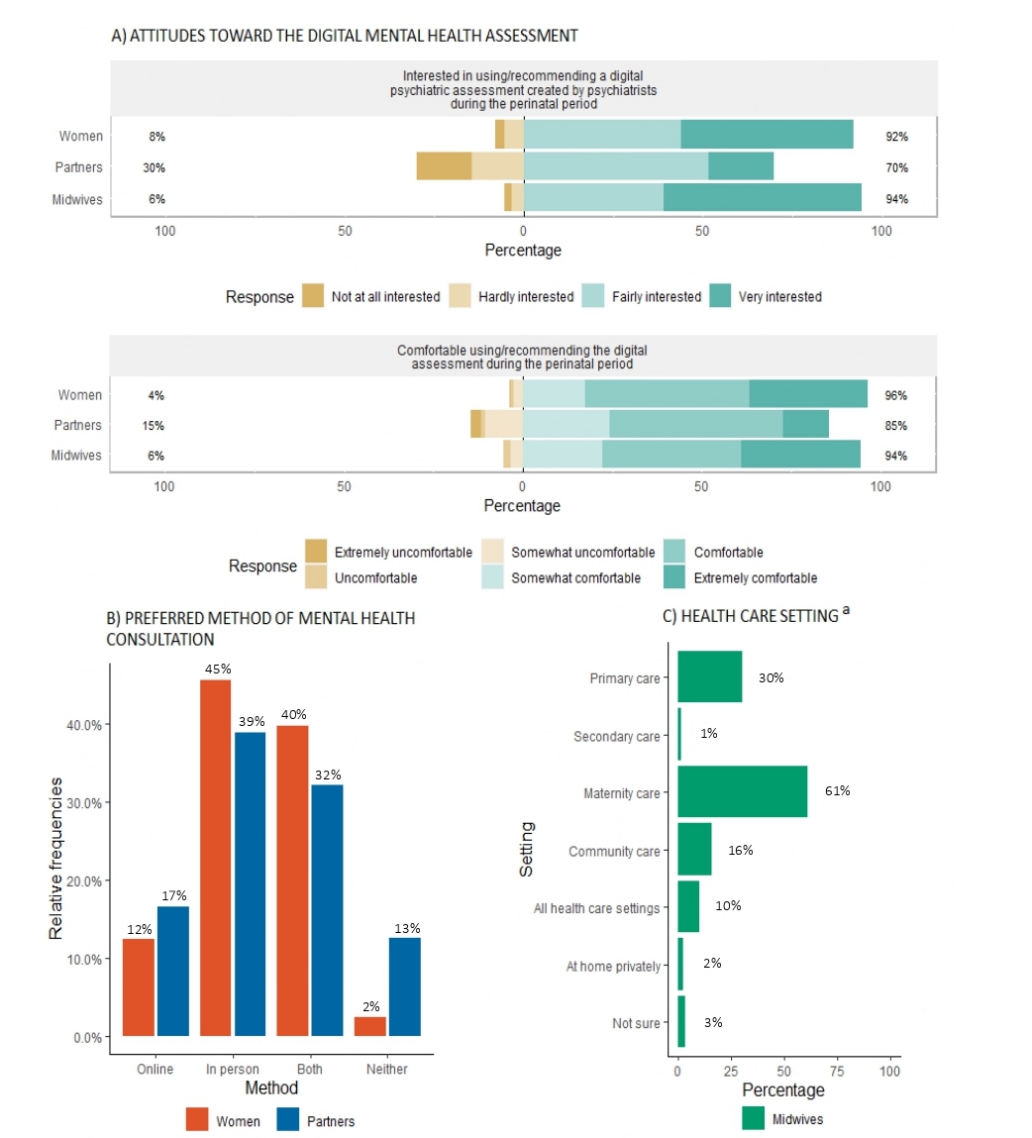
Interest in a digital mental health assessment to screen, diagnose, and triage perinatal mental health symptoms, including respondents’ interest in using or recommending a digital mental health assessment throughout the perinatal period (A), women’s and partners’ preferred method of mental health consultation following a suggestion by the digital mental health assessment that a follow-up assessment is recommended (B), and midwives’ responses as to where they see the digital mental health assessment working best (C). a: Percentages add to more than 100% as some midwives suggested more than one health care setting.

Overall, there was a strong interest in using or recommending a digital mental health assessment among women and midwives, respectively, and the majority of respondents (n=781, 76.42%) expressed that they would feel comfortable or very comfortable using or recommending a digital mental health assessment (Figure 6A). The majority of women and partners showed a preference for in-person consultations (n=417, 44.74%), followed by a blended care approach (ie, both in-person and online consultations) (n=362, 38.84%), with fewer patients preferring online-only consultations (n=120, 12.88%) (Figure 6B). The vast majority of midwives (n=55, 61.11%) reported seeing the digital mental health assessment being best placed in maternity care settings (ie, antenatal and postnatal care) (Figure 6C).

### Benefits and Barriers to Using a Digital Mental Health Assessment to Screen, Diagnose, and Triage Perinatal Mental Health Symptoms

The thematic analysis revealed 14 benefits and 17 barriers to using a digital mental health assessment to screen, diagnose, and triage perinatal mental health symptoms (see Table S1 in Multimedia Appendix 3 for initial codes, including how these were condensed into broad themes). Themes were then mapped onto the components of the COM-B model and are described below. Descriptive statistics and between-group comparisons per theme can be found in Table 1.

**Table 1:** Frequencies and between-group comparisons of the identified benefits and barriers to using a digital mental health assessment to screen, diagnose, and triage perinatal mental health symptoms.

COM-B^a^ framework, components, and themes					Frequencies (n, %)^b^									*P* value^c^				Post hoc^d^ analysis	
					Overall		Women		Partners		Midwives								
**Benefits**																			
	**Capability**																		
		**Physical capability**																	
			Accuracy	42 (4.22)		34 (4.18)		5 (5.38)		3 (3.37)		.82				—^e^			
			Usability	102 (10.24)		78 (9.58)		12 (12.90)		12 (13.48)		.31				—			
		**Psychological capability**																	
			Cognitive skills	78 (7.83)		52 (6.39)		12 (12.90)		14 (15.73)		*.001*				P,M>W			
	**Opportunity**																		
		**Physical opportunity**																	
			Accessibility	303 (30.42)		261 (32.06)		20 (21.51)		22 (24.72)		.053				—			
			Support	193 (19.38)		139 (17.08)		18 (19.35)		36 (40.45)		*<.001*				M>W,P			
			Environment	483 (48.49)		417 (51.23)		39 (41.94)		27 (30.34)		*<.001*				W>M			
			Funding and implementation	23 (2.31)		19 (2.33)		3 (3.23)		1 (1.12)		.60				—			
		**Social opportunity**																	
			Peer support	10 (1.00)		6 (0.74)		2 (2.15)		2 (2.25)		.14				—			
			Normalization of mental health	93 (9.34)		84 (10.32)		3 (3.23)		6 (6.74)		*.05*				W>P			
	**Motivation**																		
		**Automatic motivation**																	
			Positive affect	204 (20.48)		176 (21.62)		14 (15.05)		14 (15.73)		.19				—			
		**Reflective motivation**																	
			Control	73 (7.33)		57 (7.00)		5 (5.38)		11 (12.36)		.15				—			
			Reassurance	45 (4.52)		36 (4.42)		7 (7.53)		2 (2.25)		.24				—			
			Honesty	55 (5.52)		43 (5.28)		3 (3.23)		9 (10.11)		.13				—			
			Beliefs	10 (1.00)		5 (0.61)		(0) 0.00		5 (5.62)		*.002*				M>W,P			
			None	2 (0.20)		1 (0.12)		0 (0.00)		1 (1.12)		.18				—			
**Barriers**																			
	**Capability**																		
		**Physical capability**																	
			Accuracy	98 (10.25)		88 (11.41)		7 (7.29)		3 (3.37)		*.03*				W>M			
			Data protection	56 (5.86)		47 (6.10)		4 (4.17)		5 (5.62)		.84				—			
			Usability	25 (2.62)		20 (2.59)		3 (3.13)		2 (2.25)		.93				—			
		**Psychological capability**																	
			Knowledge	85 (8.89)		66 (8.56)		13 (13.54)		6 (6.74)		.21				—			
			Cognitive skills	168 (17.57)		123 (15.95)		17 (17.71)		28 (31.46)		*.002*				M>W,P			
			Technical skills	43 (4.50)		33 (4.28)		4 (4.17)		6 (6.74)		.51				—			
	**Opportunity**																		
		**Physical opportunity**																	
			Accessibility	180 (18.83)		138 (17.90)		10 (10.42)		32 (35.96)		*<.001*				M>W,P			
			Support	25 (2.62)		21 (2.72)		0 (0.00)		4 (4.49)		.10				—			
			Environment	198 (20.08)		154 (19.97)		20 (20.83)		18 (20.22)		.98				—			
			Funding and implementation	76 (7.95)		54 (7.00)		11 (11.46)		11 (12.36)		.08				—			
		**Social opportunity**																	
			Stigma	43 (4.50)		37 (4.80)		2 (2.08)		4 (4.49)		.56				—			
	**Motivation**																		
		**Automatic motivation**																	
			Negative affect	27 (2.82)		24 (3.11)		1 (1.04)		2 (2.25)		.62				—			
		**Reflective motivation**																	
			Control	66 (6.90)		55 (7.13)		8 (8.33)		3 (3.37)		.36				—			
			Beliefs	46 (4.81)		35 (4.54)		10 (10.42)		1 (1.12)		*.01*				P>W,M			
			Dishonesty	34 (3.56)		32 (4.15)		1 (1.04)		1 (1.12)		.17				—			
			Impersonal	126 (13.18)		106 (13.75)		11 (11.46)		9 (10.11)		.62				—			
			Reluctance	25 (2.62)		14 (1.82)		7 (7.29)		4 (4.49)		*.005*				P>W			
			None	60 (6.28)		47 (6.10)		8 (8.33)		5 (5.62)		.65				—			

^a^COM-B: Capability, Opportunity, and Motivation Behavior model.

^b^Sample sizes varied for the benefits and barriers due to nonresponses (ie, no response was provided). Percentages were calculated based on the total number of responses, including no identified benefits or barriers (labeled as “none”). Benefits: overall (n=996), women (n=814), partners (n=93), midwives (n=89). Barriers: overall (n=956), women (n=771), partners (n=96), midwives (n=89).

^c^*P* values are based on the Fisher exact test. Italics indicates significant values.

^d^Pairwise comparisons; W: women, P: partners, M: midwives.

^e^Not applicable.

#### Physical Capability

Overall, just under 5% (n=42) of respondents described the digital mental health assessment as having the potential to provide more accurate diagnoses by facilitating clinical decision making and being more comprehensive and objective than the current standard of care. On the other hand, the complexity of mental health and the fact that a remote approach would impede users from delving deeper into particular symptoms or concerns were seen as issues that could affect the accuracy of the tool, with significantly more women (n=88, 11.41%) than midwives (n=3, 3.37%) identifying this as a barrier. Overall, usability (ie, how user-friendly the tool is to use) was also seen as a benefit (n=102, 10.24%) and a barrier (n=25, 2.59%), while just under 6% (n=56) of all respondents saw issues surrounding data protection as barriers.

#### Psychological Capability

Cognitive skills were regarded as both a benefit and a barrier. For instance, respondents saw the potential for increased awareness and understanding of mental health as a benefit to using a digital mental health assessment, with partners (n=12, 12.90%) and midwives (n=14, 15.73%) identifying this as a benefit to a significantly greater extent in comparison to women (n=52, 6.39%). On the other hand, one’s capacity for self-reflection and awareness of difficulties, as well as general issues surrounding comprehension, were seen as barriers, with a significantly higher proportion of midwives (n=28, 31.46%) expressing these concerns relative to both the women (n=123, 15.95%) and partner (n=17, 17.71%) groups. Another barrier that was identified by approximately 9% (n=85) of all respondents regarded knowledge concerning the tool’s existence, including a general lack of awareness regarding its usefulness. Furthermore, just under 5% (n=43) of all respondents regarded poor technical skills as a barrier to using a digital mental health assessment.

#### Social Opportunity

The normalization of mental health was regarded as a benefit to using the tool, with significantly more women (n=84, 10.32%) identifying this as a benefit relative to partners (n=3, 3.23%). Peer support, including the potential to connect with other users or to share one’s experience with one’s partner and/or family, was seen as a benefit seen by a small number of respondents (n=10, 1.00%). On the other hand, stigma surrounding the use of a digital mental health assessment was seen as a potential barrier by just under 5% (n=43) of all respondents.

#### Physical Opportunity

Accessibility was seen as both a benefit and a barrier. For instance, improved access to mental health care, including it being wide-reaching and inclusive, was regarded a benefit by 30.42% (n=303) of all respondents. On the contrary, living in rural or deprived areas, as well as not having access to technology and/or the internet was seen as a barrier, with midwives (n=32, 35.96%) expressing these concerns to a significantly greater extent than women (n=138, 17.90%) and partners (n=10, 10.42%). Support was identified as a benefit and a barrier. Regarding the former, mental health care support, whether that be in contrast or in addition to the current standard of care, was regarded a benefit by respondents, with a significantly higher proportion of midwives (n=36, 40.45%) perceiving this as a benefit relative to both women (n=139, 17.08%) and partners (n=18, 19.35%). However, just under 3% (n=25) of all respondents highlighted issues surrounding the potential quality of the support offered by the tool, as well as there needing to be appropriate follow-up care, particularly given the lack of current support and available services.

Overall, almost 50% (n=483) of respondents identified environmental opportunities, such as increased convenience, flexibility, and being able to complete the assessment in a comfortable environment, as benefits. Relative to midwives (n=27, 30.34%), a significantly higher proportion of women (n=417, 51.23%) regarded environmental opportunities as benefits. On the other hand, approximately 20% (n=198) of all respondents saw not having time or a comfortable and private environment where they felt safe as barriers to using the tool. Finally, funding and implementation were seen as both a benefit and a barrier. Around 2% (n=23) of all respondents identified the use of a digital mental health assessment as being cost-effective and resulting in reduced pressure on the NHS. On the contrary, issues surrounding costs to develop and implement the tool, including its integration into the health care system, and problems associated with bureaucracy and infrastructure in the NHS, were identified as barriers by almost 8% (n=76) of all respondents.

#### Automatic Motivation

Positive affect was identified as a benefit to using a digital mental health assessment. In particular, 20.48% (n=204) of all respondents saw the tool being less intimidating and intrusive, as well as less stressful to use, resulting in reduced anxiety relative to in-person care. On the contrary, negative affect was seen as a barrier, with issues surrounding the tool creating more distress and a general fear of the results being identified by approximately 3% (n=27) of all respondents.

#### Reflective Motivation

Control of one’s own mental health was reported as both a benefit and a barrier. Feeling empowered and being in charge of one’s own mental health were regarded as benefits by 7.33% (n=73) of all respondents. On the other hand, 6.90% (n=66) of all respondents also perceived having to take the initiative to prioritize one’s mental health as a barrier. Overall, approximately 4% (n=34) of all respondents saw it being easier to manipulate answers in order to obtain a certain outcome, as well as general issues surrounding dishonesty, as barriers. However, almost 6% (n=55) of all respondents also saw the potential to be more honest regarding mental health symptoms as a benefit.

In addition, reassurance, including feeling cared for and heard, was also mentioned as a benefit by almost 5% (n=45) of respondents, while issues surrounding the tool being impersonal were identified as barriers by around 13% (n=126) of respondents. Beliefs about the tool were seen as both benefits and barriers to its use. For instance, beliefs regarding the tool being trustworthy and evidence-based, as well as having the potential to revolutionize mental health care provision, were seen as benefits, with a significantly higher proportion of midwives (n=5, 5.62%) identifying this in comparison to the women (n=5, 0.61%) and partner (0%) groups. On the contrary, skepticism regarding the credibility of the tool and beliefs associated with a digital tool being inappropriate to diagnose mental health conditions were identified as barriers, with partners (n=10, 10.42%) expressing this concern to a significantly greater extent than women (n=35, 4.54%) and midwives (n=1, 1.12%). Finally, a general reluctance to using the tool, including being resistant to change, was seen as a barrier, with a significantly higher proportion of partners (n=7, 7.29%) identifying this relative to the women (n=14, 1.82%).

## Discussion

### Principal Findings

The ultimate goal of this research was to provide proof-of-concept support for the development and implementation of a digital mental health assessment to aid in the identification and triaging of perinatal mental health concerns in the United Kingdom. First, we explored the current state of perinatal mental health care provision, as well as in the context of the evolving COVID-19 pandemic, relative to NICE guidelines [1]. Second, we evaluated women’s, partners’, and midwives’ attitudes toward using a digital mental health assessment to screen, diagnose, and triage perinatal mental health concerns.

The vast majority of women and one-third of partners expressed having experienced poor mental health during the perinatal period, with the most typical conditions encountered by midwives throughout their careers being depression, anxiety, and PTSD. Critically, and in line with previous findings, rates of diagnoses were low relative to the number of individuals reporting concerns (eg, [29-31]). Perhaps more alarmingly, however, was the fact that the majority of respondents had referred themselves to an HCP, rather than being identified by a midwife as requiring further evaluation. Importantly, it has been shown that midwives may struggle to recognize perinatal mental health difficulties and are less inclined to refer women to a mental health specialist for further evaluation relative to other maternity HCPs [32]. This may be due, in part, to the low uptake of validated mental health screening tools in maternity care settings. Despite NICE guidelines [1] recommending the use of the Whooley Questions [33] and the 2‑item Generalized Anxiety Disorder scale (GAD‑2) [34] to screen for perinatal mental health problems, 60% of midwives in this study reported *not* using standardized questions or questionnaires.

Mental health care support following a miscarriage or termination or difficult birth was also largely overlooked, particularly when it came to partners. In fact, the vast majority of partners were not provided with any information or support throughout the perinatal period. This is in line with past research suggesting that partners often feel excluded in favor of a more women-centric approach to perinatal care [35-37]. Importantly, despite maternity care settings being women centric, the routine assessment of women’s past or family history of mental health difficulties was poor and varied considerably across midwives. This presents a significant issue as the majority of women in this study expressed having had the condition *prior* to becoming pregnant. Indeed, women with a past history of mental disorders are often at risk of relapse and may need support with re‐emerging symptoms precipitated by pregnancy or postpartum [38-40]. NICE guidelines [1] recommend that *all* women should be routinely asked about past or present severe mental health conditions and treatment, as well as the presence of severe mental health in first-degree relatives. However, our findings indicate that, overall, these recommendations are not being followed in maternity care settings in the United Kingdom. This may be due to the many tasks that are normally performed during appointments, lack of training, discontinuity of care, and time constraints [41].

Critically, the COVID-19 pandemic has put additional pressure on midwives and has had a devastating impact on the mental health of patients. Over 60% of midwives reported having seen an increase in the number of women experiencing perinatal mental health difficulties. This was mirrored by the women, with 64% reporting poorer mental health since the outbreak. In contrast with these alarming figures, less than one-quarter of women had been asked about the effects of the pandemic on their mental health, and the majority of midwives reported there being no change in the extent to which mental health had been prioritized throughout the pandemic. However, this was closely followed by there being a greater emphasis on inquiring about mental health, highlighting inconsistencies in care provision across maternity care settings. Notably, midwives reported there being a general lack of guidance on how to best support women’s perinatal mental health throughout the pandemic. Notwithstanding the recommendations by the Royal College of Obstetricians and Gynaecologists [13] on the use of remote means throughout the pandemic, only half of midwives reported using remote tools to support the needs of women who may be at risk of perinatal mental health difficulties, with less than one-third of women having discussed their mental health concerns with a midwife or HCP remotely.

Despite the low use of remote means to support perinatal mental health difficulties throughout the pandemic, there was a strong interest in using a digital mental health assessment to screen, diagnose, and triage perinatal mental health concerns, particularly among women and midwives. The digital assessment was seen to be well placed within maternity care settings, with an in-person only or a blended approach (ie, a combination of in-person and remote support) being preferred by women and partners in the event of further care being advised. The results of our COM-B analysis highlighted important implications for the development and implementation of a digital mental health assessment. Physical opportunities, such as increased convenience and flexibility relative to the current standard of care, were seen as some of the key benefits to using a perinatal digital mental health assessment, particularly among women. This result is in agreement with previous findings reported by a systematic literature review focused on digital health for perinatal care, which highlighted that patients saw the convenience of receiving care in their homely ambience as a benefit of digital interventions, with satisfaction rates varying between 86% and 95% in digital mental health studies and 90% in electronically monitored induced home births [42].

Increased accessibility to mental health care provision, including the potential for improved parity of care, was also reported as a benefit by the participants of our study. Importantly, accessibility was also regarded as a potential barrier to using the tool, especially among midwives, who expressed concerns regarding accessing care in remote or rural locations with poor internet connectivity, as well as issues surrounding the inclusion of individuals from a low socioeconomic background. Our findings on the theme of accessibility are in line with previous research on attitudes of women and health care professionals toward broadly defined mobile health interventions during pregnancy [43]. Mobile and digital technologies were perceived as a useful tool to increase access to care for at-risk women who could not attend perinatal clinics, but also as a potential source of digital exclusion among those who are socioeconomically disadvantaged.

Consequently, the perinatal digital mental health assessment should be designed to leverage perceived physical opportunity benefits while mitigating barriers. For instance, flexibility in terms of assessment completion and the option to complete the assessment in the comfort of one’s home may increase engagement. Similarly, interoperability with various devices, the possibility to store assessment responses in the absence of good connectivity, or the possibility to conduct the assessment via text messaging may increase accessibility in hard-to-reach groups. Text messaging has often proved to be a suitable tool for the delivery of mental health services to rural and remote communities, and future research in this field should focus on improving the predictive models and computational linguistic tools that underlie the diagnostic and therapeutic value of text-based psychological services [44].

Positive affect (automatic motivation component) was also readily recognized as a benefit. Women, partners, and midwives highlighted positive emotions related to using a digital assessment, such as reduced anxiety and stress, and an increased sense of privacy. On the other hand, the perception of the assessment being impersonal (reflective motivation component) was commonly highlighted as a barrier. Thus, a personalized digital journey and the possibility of a follow-up assessment by an HCP may maintain the positive feelings of privacy while addressing the perceived lack of in-person care. Lastly, barriers were identified in relation to cognitive skills (psychological capability component), such as comprehension difficulties, poor self-reflection, and difficulties in expressing and acknowledging mental health issues. This was particularly concerning for midwives.

Therefore, it is crucial to design a tool that can cater to individuals who may find it difficult to complete a digital mental health assessment, such as those with poor comprehension skills, learning difficulties, or mental health symptoms that impair communication. This point was also raised in the research priority setting exercise coordinated by the James Lind Alliance Priority Setting Partnership for the “Digital Technology for Mental Health: Asking the Right Questions” project [44. The uncertainty around how mental health conditions can affect engagement with digital technologies was identified by patients, carers, and mental health care providers as a research question that must be addressed. Adopting a co-designing approach to development, where all potential users (eg, women and their families, midwives, mental health specialists) and stakeholders are regarded as collaborators, could help overcome barriers related to cognitive skills by ensuring that the product meets users’ needs and preferences whilst being clinically valid and feasible to deliver.

Taken together, the findings from this study highlight the urgent need to improve and standardize perinatal mental health care provision in the United Kingdom. While time is limited in maternity care settings, where midwives may feel pressured to prioritize physical over mental health, our study provides proof-of-concept support for the use of a digital mental health assessment tool as an innovative time- and cost-effective solution to the identification and treatment of perinatal mental health concerns. Digital technologies have the potential to support midwives in the recognition of perinatal mental health difficulties and are highly scalable. This means that support can be provided to the *family unit* as a whole, resulting in a comprehensive and scalable approach to the provision of mental health care throughout an often challenging time for women and their families.

### Strengths and Limitations

The surveys were comprehensive and were carefully designed with input from an experienced perinatal psychiatrist and a practicing midwife. Furthermore, the use of mixed methods allowed for the revelation of information that would have not been obtained via quantitative research only. Additionally, the use of COM-B, a comprehensive supratheory model, helped develop a map of the broad landscape of behavior determinants that can be considered for the implementation of a digital mental health assessment. Patients’ and HCPs’ behaviors are often considered separately in behavior change literature. However, this introduces additional levels of complexity and the need for different models to understand a single intervention. The COM-B model, along with our choice of exploring both users’ and midwives’ views, offers a single comprehensive framework incorporating context and stakeholders that can be used to inform the design of future digital perinatal mental health assessment interventions.

Critically, the majority of the women and partners who participated in this study were highly educated and had an above average socioeconomic status, meaning that the findings from this study may not be generalizable to the broader UK population. Furthermore, individuals with mental health concerns and/or negative experiences with mental health care provision may have been more receptive to the recruitment materials and more likely to enroll in the study. As such, there is likely to be a recruitment bias. In addition, women and midwives were recruited separately and, as a consequence, associations between care provision and care experience should be interpreted with caution.

## Appendix

Multimedia Appendix 1 Women and partners: sociodemographic and perinatal health characteristics, mental health provision, symptoms, and diagnosis, and COVID-19.

Multimedia Appendix 2 Midwives: sociodemographic characteristics, mental health provision, and COVID-19.

Multimedia Appendix 3 Benefits and barriers listed by codes and grouped by theme, COM-B component, and framework.

## Abbreviations

COM-B model: Capability, Opportunity, and Motivation Behavior model
GAD: Generalized Anxiety Disorder
HCP: health care professional
NHS: National Health Service
NICE: National Institute for Health and Care Excellence
PHQ-9: Patient Health Questionnaire-9
PTSD: posttraumatic stress disorder
