# Supplementary material for: Proof-of-Concept Support for the Development and Implementation of a Digital Assessment for Perinatal Mental Health: Mixed Methods Study
Source: J Med Internet Res. 2021 Jun 4;23(6):e27132. doi: 10.2196/27132 (PMC8183599; doi:10.2196/27132)
Supplement: Multimedia Appendix 1 [file jmir_v23i6e27132_app1.docx]

**Multimedia Appendix 1**

**Supplementary Table 1.** *Women and partners: socio-demographic and perinatal health characteristics, mental health provision, symptoms, and diagnosis, and COVID-19*

|  |  | **Women (*N* = 829)** | **Partners (*N* = 103)** |
| --- | --- | --- | --- |
| 1. **Socio-demographic characteristics** | | ***M* (SD)** | |
| Age |  | 31.78 (4.63) | 34.87 (6.38) |
|  |  | ***n* (%)** | |
| Ethnicity | White | 784 (94.57) | 88 (85.44) |
|  | Asian/Asian British | 19 (2.29) | 6 (5.83) |
|  | Black/African/Caribbean/Black British | 12 (1.48) | 3 (2.91) |
|  | Mixed/Multiple ethnic groups | 12 (1.48) | 4 (3.88) |
|  | Arab | 2 (0.24) | 2 (1.94) |
| Education | Below GCSE/equivalent | 3 (0.36) | - |
|  | GCSE/Scottish higher/equivalent | 29 (3.50) | 13 (12.62) |
|  | A Level/IB/Advanced higher | 110 (13.27) | 13 (12.62) |
|  | Undergraduate degree | 379 (45.72) | 45 (43.69) |
|  | Postgraduate degree | 308 (37.15) | 32 (31.07) |
| Relationship/living arrangement | Single | 19 (2.29) | 2 (1.94) |
|  | Married/Civil partnership | 548 (66.10) | 78 (75.73) |
|  | Cohabiting | 247 (29.79) | 21 (20.39) |
|  | Separated | 4 (0.48) | 2 (1.94) |
|  | Other | 11 (1.33) | - |
| Accommodation | Rented accommodation | 180 (21.71) | 21 (20.39) |
|  | Own house | 585 (70.57) | 74 (71.84) |
|  | Family home | 38 (4.58) | 4 (3.89) |
|  | Other | 26 (3.14) | 4 (3.89) |
| Employment | Employed | 501 (60.43) | 87 (84.47) |
|  | Self-employed | 41 (4.95) | 11 (10.68) |
|  | Parental leave | 231 (27.86) | 1 (0.97) |
|  | Student | 19 (2.29) | 2 (1.94) |
|  | Unemployed | 35 (4.22) | 2 (1.94) |
| Household income | Less than £15,000 | 25 (3.02) | 1 (0.97) |
|  | £15,001 - £ 25,000 | 66 (7.96) | 6 (5.83) |
|  | £25,001 - £35,000 | 90 (10.86) | 8 (7.77) |
|  | £35,001 - £45,000 | 128 (15.44) | 13 (12.62) |
|  | £45,001 - £55,000 | 128 (15.44) | 15 (14.56) |
|  | £55,001 - £65,000 | 99 (11.94) | 17 (16.50) |
|  | £65,001 - £75,000 | 87 (10.49) | 12(11.65) |
|  | £75,001 - £85,000 | 65 (7.84) | 10 (9.71) |
|  | More than £85,001 | 130 (15.68) | 20 (19.42) |
|  | Prefer not to answer | 11 (1.33) | - |
| 1. **Perinatal health characteristics** | | | |
| Pregnancy status | Planning/trying to conceive | 76 (9.17) | 11 (10.68) |
|  | 1^st^ trimester | 43 (5.19) | 6 (5.83) |
|  | 2^nd^ trimester | 112 (13.51) | 18 (17.48) |
|  | 3^rd^ trimester | 104 (12.55) | 14 (13.59) |
|  | 0-3 months after giving birth | 196 (23.64) | 23 (22.33) |
|  | 3-6 months after giving birth | 128 (15.44) | 12 (11.65) |
|  | 6 months-1 year after giving birth | 124 (14.96) | 16 (15.53) |
|  | 1-2 years after giving birth | 46 (5.56) | 3 (2.91) |
| Child number | 1^st^ child | 582 (70.21) | 78 (75.73) |
|  | 2^nd^ child | 184 (22.20) | 19 (18.45) |
|  | 3^rd^ child | 34 (4.10) | 2 (1.94) |
|  | 4^th^ child | 11 (1.33) | 3 (2.91) |
|  | Other | 18 (2.17) | 1 (0.97) |
| Time to conception | Have not started trying yet | 29 (3.50) | 4 (3.88) |
|  | Less than 3 months | 347 (41.86) | 40 (38.83) |
|  | Less than 9 months | 159 (19.18) | 15 (14.56) |
|  | About 1 year | 59 (7.12) | 9 (8.74) |
|  | More than 1 year | 136 (16.41) | 19 (18.45) |
|  | Not sure | 5 (0.60) | 4 (3.88) |
|  | Unplanned pregnancy | 93 (11.22) | 12 (11.65) |
| Health care^a^ | NHS health care | 725 (92.35) | 81 (87.10) |
|  | NHS and private health care | 55 (7.01) | 11 (11.83) |
|  | Private health care | 5 (0.64) | 1 (1.08) |
| Fertility treatment^b^ | Yes | 73 (10.33) | 11 (12.64) |
|  | Not yet, but planning to | 4 (0.57) | - |
|  | No | 630 (89.11) | 76 (87.36) |
| Termination of pregnancy (e.g., abortion, ectopic pregnancy, other medical intervention)^b^ | Yes | 49 (6.93) | 14 (16.09) |
|  | No | 654 (92.50) | 73 (83.91) |
|  | Prefer not to answer | 4 (0.57) | - |
| Miscarriage^b^ | Yes | 157 (22.21) | 16 (18.39) |
|  | No | 544 (76.94) | 71 (81.61) |
|  | Prefer not to answer | 6 (0.85) | - |
| Difficult birth/complications^c^ | Yes | 262 (53.04) | 26 (48.15) |
|  | No | 232 (46.96) | 27 (50.00) |
|  | Not sure | - | 1 (1.85) |
| 1. **Mental health provision** | | | |
| Information about mental health provided at any pregnancy/birth-related appointment^a^ | Yes | 576 (73.38) | 15 (16.13) |
|  | No | 209 (26.62) | 78 (83.87) |
| Mental health information provided via^dα^ | Face-to-face during appointment(s) | 480 (86.24) | 10 (66.67) |
|  | Leaflets | 356 (61.81) | 11 (73.33) |
|  | Web resources (e.g., NHS website) | 100 (17.36) | 3 (20.00) |
|  | Applications/other digital tools | 41 (7.12) | 2 (13.33) |
|  | Other | 32 (5.56) | 2 (13.33) |
| Was asked about mood/mental health during pregnancy^e^ | Yes | 637 (84.59) | 11 (11.96) |
|  | No | 116 (15.41) | 69 (75.00) |
|  | NA | - | 12 (13.04) |
| Was asked about mood/mental health after birth^c^ | Yes | 456 (92.31) | 11 (20.37) |
|  | No | 38 (7.69) | 39 (72.22) |
|  | NA | - | 4 (7.41) |
| Mental health support/advice offered due to termination/miscarriage^f^ | Yes | 45 (28.30) | 1 (6.25) |
|  | No | 114 (71.70) | 15 (93.75) |
| Mental health support/advice offered due to difficult birth/complications^g^ | Yes | 58 (22.14) | - |
|  | No | 204 (77.86) | 1. 100) |
| 1. **Mental health symptoms and diagnosis** | | | |
| Suffered with mental health symptoms during/after pregnancy^e^ | Yes | 469 (62.28) | 30 (32.61) |
|  | No | 215 (28.55) | 49 (53.26) |
|  | Not sure | 69 (9.16) | 13 (14.13) |
| Diagnosed with a mental health condition by a HCP during/after pregnancy^h^ | Yes | 65 (13.86) | 1 (3.33) |
|  | No | 404 (86.14) | 29 (96.67) |
| Diagnosis^iβ^ | Depression | 25 (38.46) | - |
|  | Anxiety | 25 (38.46) | 1 (100) |
|  | Social anxiety | 1 (1.54) | - |
|  | Bipolar I/II | 4 (6.15) | - |
|  | Adjustment disorder | 1 (1.54) | - |
|  | PTSD | 5 (7.69) | - |
|  | OCD | 2 (4.62) | - |
|  | Personality disorder | 5 (7.69) | - |
|  | Eating disorders | 3 (4.71) | - |
|  | Tokophobia | 1 (1.54) | - |
|  | Panic disorder | 2 (4.62) | - |
|  | Agoraphobia | 1 (1.54) | - |
| Time of onset^i^ | I already had the condition before | 36 (55.38) | - |
|  | It happened during pregnancy | 8 (12.31) | 1 (100) |
|  | It happened within one year after birth | 17 (26.15) | - |
|  | Not sure | 4 (6.15) | - |
| Referral process^i^ | Was referred by a health care professional involved in pregnancy/postnatal care | 9 (13.85) | - |
|  | Self-referred | 51 (78.46) | 1 (100) |
|  | Was encourage to seek help by partner/family | 5 (7.69) | - |
| 1. **COVID-19 and mental health^Ω^** | | | |
| Poorer mental health since COVID-19^j^ | Yes | 293 (64.40) | - |
|  | No | 105 (23.08) | - |
|  | Not sure | 57 (12.57) | - |
| Was asked about the effects of COVID-19 on mood/mental health by a health care professional involved in pregnancy/postnatal period^k^ | Yes | 93 (20.85) | - |
|  | No | 353 (79.15) | - |
| Has discussed mental health symptoms remotely with a health care professional involved in pregnancy/postnatal period | Yes | 120 (26.91) | - |
|  | No | 326 (73.09) | - |

***Note.*** COVID-19, coronavirus 19; HCP, health care professional; NA, not applicable; OCD, obsessive-compulsive disorder; PTSD, post-traumatic stress disorder.
***Key.*** ^a^ Includes those in contact with health care system (women = 785; partners = 93); ^b^ Includes those who have started trying to conceive and planned pregnancies (women = 707; partners = 87); ^c^ Includes those who had given birth (women = 494; partners = 54); ^d^ Includes those who responses ‘yes’ to having been provided with information about mental health (women = 576; partners = 15). ^α^ Percentages add to more than 100% as participants could select multiple options. ^e^ Includes those who are pregnant or have given birth (women = 753; partners = 92);
^f^ Includes those who answered ‘yes’ to termination/miscarriage question (women = 159; partners = 16); ^g^ Includes those who answered ‘yes’ to birth complications question (women = 262; partners = 26); ^h^ Includes those who answered ‘yes’ to suffering with mental health symptoms during/after pregnancy (women = 469; partners = 30); ^i^ Includes those who were diagnosed with a mental health condition (women = 65; partners = 1); ^β^ Percentages add to more than 100% due to comorbidities; ^Ω^ The partner survey did not include these questions; ^j^ Includes those who are pregnant or have given birth within the last 3 months (women = 455). ^k^ Includes those who are pregnant or have given birth within the last 3 months and have been in contact with a midwife since lockdown (women = 446).
