# Supplementary material for: Proof-of-Concept Support for the Development and Implementation of a Digital Assessment for Perinatal Mental Health: Mixed Methods Study
Source: J Med Internet Res. 2021 Jun 4;23(6):e27132. doi: 10.2196/27132 (PMC8183599; doi:10.2196/27132)
Supplement: Multimedia Appendix 2 [file jmir_v23i6e27132_app2.docx]

**Multimedia Appendix 2**

**Supplementary Table 1.** *Midwives: socio-demographic characteristics, mental health provision, and COVID-19*

|  |  | **Midwives (*N* = 90)** |
| --- | --- | --- |
| 1. **Socio-demographic characteristics** | | ***M* (SD)** |
| Age |  | 39.90 (9.67) |
| Years of practise |  | 10.36 (7.19) |
|  |  | ***n* (%)** |
| Gender | Female | 89 (98.89) |
|  | Male | 1 (1.11) |
| Education | Undergraduate degree | 67 (74.44) |
|  | Postgraduate degree | 15 (16.67) |
|  | Other | 8 (8.89) |
| 1. **Mental health provision** | | ***n* (%)** |
| Provide patients with information on mental health during ante/postnatal appointments | Always (every patient) | 30 (33.33) |
|  | Often (1 out of 2 patients) | 21 (23.33) |
|  | Sometimes (1 out of 5 patients) | 20 (22.22) |
|  | Rarely (1 out of 10 patients) | 7 (7.78) |
|  | Never | 1 (1.11) |
|  | NA | 11 (12.22) |
| Mental health information provided via^aα^ | Face-to-face during appointments | 71 (91.03) |
|  | Leaflets | 31 (39.74) |
|  | Web resources (e.g., NHS website) | 21 (26.92) |
|  | Applications/other digital tools | 9 (11.54) |
|  | Other | 7 (8.97) |
| Typical tools used to screen for patients’ mental health symptoms^α^ | Whooley Questions | 28 (31.11) |
|  | GAD-7 | 15 (16.67) |
|  | PHQ-9 | 9 (10.00) |
|  | EPDS | 2 (2.22) |
|  | Birth Trauma Scale | 2 (2.22) |
|  | None | 55 (61.11) |
| Typical mental health symptoms/conditions seen throughout career^α^ | Low mood/depression | 88 (97.78) |
|  | Excessive worrying/anxiety | 89 (98.89) |
|  | Alcohol/substance abuse | 63 (70.00) |
|  | Mania/bipolar disorder | 61 (67.78) |
|  | Psychosis | 48 (53.33) |
|  | Trauma/PTSD | 83 (92.22) |
|  | Other | 15 (16.67) |
| Patients can be referred directly to a mental health specialist | Yes | 86 (95.56) |
|  | No (have to see their GP first) | 4 (4.44) |
| Aware of length of referral process | Yes | 52 (57.78) |
|  | No | 33 (36.67) |
|  | NA | 5 (5.56) |
| 1. **Partners’ mental health provision** | | |
| Provide partners with information on mental health during ante/postnatal appointments | Always (every patient) | 6 (6.67) |
|  | Often (1 out of 2 patients) | 7 (7.78) |
|  | Sometimes (1 out of 5 patients) | 23 (25.56) |
|  | Rarely (1 out of 10 patients) | 28 (31.11) |
|  | Never | 19 (21.11) |
|  | NA | 7 (7.78) |
| Mental health information provided via^bα^ | Face-to-face during appointments | 56 (87.50) |
|  | Leaflets | 20 (31.25) |
|  | Web resources (e.g., NHS website) | 11 (17.19) |
|  | Applications/other digital tools | 5 (7.81) |
| Typical mental health symptoms/conditions seen throughout career^α^ | Low mood/depression | 61 (67.78) |
|  | Excessive worrying/anxiety | 56 (62.22) |
|  | Alcohol/substance abuse | 31 (34.44) |
|  | Mania/bipolar disorder | 9 (10.00) |
|  | Psychosis | 8 (8.89) |
|  | Trauma/PTSD | 34 (37.78) |
|  | Other | 2 (2.22) |
|  | NA | 11 (12.22) |
| 1. **COVID-19 and mental health** | | |
| Increase in number of patients experiencing perinatal mental health symptoms since COVID-19 | Yes | 55 (61.11) |
|  | No | 18 (20.00) |
|  | Not sure | 13 (14.44) |
|  | NA | 4 (4.44) |
| Emphasis on assessing/discussing mental health symptoms since COVID-19 | Yes, assessing/discussing mental health symptoms is more important now | 35 (38.89) |
|  | No, assessing/discussing mental health symptoms has been deprioritised | 7 (7.78) |
|  | No change | 38 (42.22) |
|  | Not sure | 8 (8.89) |
|  | NA | 2 (2.22) |
| Emphasis on using remote means (e.g., telephone, video consultations) to support those at risk of perinatal mental health problems since COVID-19 | Yes | 47 (52.22) |
|  | No | 19 (21.11) |
|  | Not sure  NA | 22 (24.44)  2 (2.22) |
| Barriers to assessing/discussing mental health symptoms during COVID-19^α^ | Shortage of staff | 27 (30.00) |
|  | Time constraints | 35 (38.89) |
|  | Pressure to prioritise physical over mental health | 32 (35.56) |
|  | Lack of guidance | 45 (50.00) |

***Note.*** COVID-19, coronavirus 19; EPDS; Edinburgh Postnatal Depression Scale; GAD-7, Generalised Anxiety Disorder 7-item questionnaire; NA, not applicable; PHQ-9, Patient Health Questionnaire-9; PTSD, post-traumatic stress disorder.
***Key.*** ^a^ Includes those who did not answer ‘never’ or ‘NA’ to providing patients with information on mental health symptoms (*n* = 78); ^α^ Percentages add to more than 100% as participants could select multiple options; ^b^ Includes those who did not answer ‘never’ or ‘NA’ to providing partners with information on mental health symptoms (*n* = 64).
