# Supplementary material for: Proof-of-Concept Support for the Development and Implementation of a Digital Assessment for Perinatal Mental Health: Mixed Methods Study
Source: J Med Internet Res. 2021 Jun 4;23(6):e27132. doi: 10.2196/27132 (PMC8183599; doi:10.2196/27132)
Supplement: Multimedia Appendix 3 [file jmir_v23i6e27132_app3.docx]

**Multimedia Appendix 3**

**Supplementary Table 1.** *Benefits and barriers listed by codes and grouped by theme, COM-B component and framework.*

| **Benefits** | | | |
| --- | --- | --- | --- |
|  | | **COM-B** | |
| **Codes** | **Themes** | **Components** | **Framework** |
| Aid in clinical decision-making | *Accuracy* | **Physical capability** | **Capability** |
| Comprehensive |  |  |  |
| Early identification |  |  |  |
| Focused assessment |  |  |  |
| More objective |  |  |  |
| Ease | *Usability* |  |  |
| Can think about responses | *Cognitive skills* | **Psychological capability** |  |
| Easier to open up |  |  |  |
| Increased awareness/understanding of MH |  |  |  |
| Accessibility | *Accessibility* | **Physical opportunity** | **Opportunity** |
| Equal treatment |  |  |  |
| Hard-to-reach groups |  |  |  |
| Wide-reaching/inclusivity |  |  |  |
| Comfortable | *Environment* |  |  |
| Convenient |  |  |  |
| Flexibility |  |  |  |
| No need to make/wait for an appointment |  |  |  |
| Pandemic |  |  |  |
| Time |  |  |  |
| Cost-effective | *Funding and implementation* |  |  |
| Digital record |  |  |  |
| Reduced pressure on NHS |  |  |  |
| Extended/enhanced care | *Support* |  |  |
| Individualised approach |  |  |  |
| Initial step |  |  |  |
| Monitoring throughout perinatal period |  |  |  |
| Support |  |  |  |
| Timely support |  |  |  |
| Connect with other mums | *Peer support* | **Social opportunity** |  |
| Share experience with partner/family |  |  |  |
| Normalization of MH | *Normalization of mental health* |  |  |
| Reduced fear of judgement |  |  |  |
| Reduced shame |  |  |  |
| Reduced stigma |  |  |  |
| Less awkward | *Positive affect* | **Automatic motivation** | **Motivation** |
| Less formal |  |  |  |
| Less intimidating |  |  |  |
| Less intrusive |  |  |  |
| Less pressure |  |  |  |
| Less stressful |  |  |  |
| No face to face contact |  |  |  |
| Privacy/anonymity |  |  |  |
| Reduced anxiety |  |  |  |
| Confidence | *Control* | **Reflective motivation** |  |
| Control of own MH |  |  |  |
| Less effort required |  |  |  |
| Feeling cared for/heard | *Reassurance* |  |  |
| Not wasting professional's time |  |  |  |
| Validation/reassurance |  |  |  |
| Honesty | *Honesty* |  |  |
| Official tool | *Beliefs* |  |  |
| Revolutionary |  |  |  |
| **Barriers** | | | |
|  | | **COM-B** | |
| **Codes** | **Themes** | **Components** | **Framework** |
| Accuracy | *Accuracy* | **Physical capability** | **Capability** |
| Can't delve deeper into particular symptoms |  |  |  |
| Complexity of MH |  |  |  |
| Fluctuations in symptoms |  |  |  |
| Quality of assessment/questions |  |  |  |
| Subtle cues (including body language and vocal cues) |  |  |  |
| Data protection/confidentiality/security | *Data protection* |  |  |
| Privacy |  |  |  |
| Length of assessment | *Usability* |  |  |
| Usability |  |  |  |
| Awareness | *Knowledge* | **Psychological capability** |  |
| Knowledge of benefits/perceived usefulness |  |  |  |
| Knowledge regarding the assessment |  |  |  |
| Knowledge by HCPs |  |  |  |
| Awareness of difficulties | *Cognitive skills* |  |  |
| Capacity for self-reflection |  |  |  |
| Comprehension/language |  |  |  |
| Difficulty expressing yourself |  |  |  |
| Difficulty opening up |  |  |  |
| Engagement |  |  |  |
| Mental health symptoms |  |  |  |
| Mindset/concentration |  |  |  |
| Overwhelmed by information |  |  |  |
| Subjectivity |  |  |  |
| Technological literacy | *Technical difficulties* |  |  |
| Accessibility | *Accessibility* | **Physical opportunity** | **Opportunity** |
| Availability |  |  |  |
| Appropriate follow-up care | *Support* |  |  |
| Quality of support |  |  |  |
| Safeguarding |  |  |  |
| Comfortable environment | *Environment* |  |  |
| Technical issues |  |  |  |
| Time |  |  |  |
| Competition | *Funding & implementation* |  |  |
| Cost |  |  |  |
| Cost to develop/implement |  |  |  |
| NHS infrastructure |  |  |  |
| Resources |  |  |  |
| Stand-alone tool (not integrated into health care system) |  |  |  |
| Fear of disclosure | *Stigma* | **Social opportunity** |  |
| Fear of judgement |  |  |  |
| Labelling |  |  |  |
| Stigma |  |  |  |
| Could create more distress | *Negative affect* | **Automatic motivation** | **Motivation** |
| Device time on MH |  |  |  |
| Fear of results |  |  |  |
| Guilt |  |  |  |
| Shame |  |  |  |
| Acceptance of difficulties | *Control* | **Reflective motivation** |  |
| Confidence |  |  |  |
| Control of own MH |  |  |  |
| Motivation |  |  |  |
| Credibility | *Beliefs* |  |  |
| Diagnosis via app is inappropriate |  |  |  |
| Not an official assessment |  |  |  |
| Hiding emotions | *Dishonesty* |  |  |
| Honesty |  |  |  |
| Missing in-person element | *Impersonal* |  |  |
| Novelty | *Reluctance* |  |  |
| Reluctance by patient & HCPs |  |  |  |

***Key.*** HCP = Health care professional, MH = Mental health, NHS = National Health Service
